# On-Surface Bottom-Up Construction of COF Nanoshells towards Photocatalytic H_2_ Production

**DOI:** 10.34133/2021/9798564

**Published:** 2021-08-02

**Authors:** Yao Chen, Dong Yang, Yuchen Gao, Runlai Li, Ke An, Wenjing Wang, Zhanfeng Zhao, Xin Xin, Hanjie Ren, Zhongyi Jiang

**Affiliations:** ^1^Key Laboratory for Green Chemical Technology, School of Chemical Engineering and Technology, Tianjin University, Tianjin 300072, China; ^2^Collaborative Innovation Center of Chemical Science and Engineering (Tianjin), Tianjin 300072, China; ^3^Key Laboratory of Systems Bioengineering of Ministry of Education, School of Chemical Engineering and Technology, Tianjin University, Tianjin 300072, China; ^4^School of Environmental Science and Engineering, Tianjin University, Tianjin 300072, China; ^5^College of Polymer Science and Engineering, Sichuan University, Chengdu 610065, China; ^6^Joint School of National University of Singapore and Tianjin University, International Campus of Tianjin University, Binhai New City, Fuzhou 350207, China

## Abstract

The rational design of an outer shell is of great significance to promote the photocatalytic efficiency of core-shell structured photocatalysts. Herein, a covalent organic framework (COF) nanoshell was designed and deposited on the cadmium sulfide (CdS) core surface. A typical COF material, TPPA, featuring exceptional stability, was synthesized through interfacial polymerization using 1, 3, 5-triformylphloroglucinol (TP) and p-phenylenediamine (PA) as monomers. The nanoshell endows the CdS@TPPA nanosphere with ordered channels for unimpeded light-harvesting and fast diffusion of reactants/products and well-defined modular building blocks for spatially charge separation. Moreover, the heterojunction formed between CdS and TPPA can further facilitate the effective charge separation at the interface *via* lower exciton binding energy compared with that of pristine TPPA. By modulating the thickness of TPPA nanoshell, the CdS@TPPA nanosphere photocatalyst with the nanoshell thickness of about 8 ± 1 nm exhibits the highest photocatalytic H_2_ evolution of 194.1 *μ*mol h^−1^ (24.3 mmol g^−1^ h^−1^, 8 mg), which is superior to most of the reported COF-based photocatalysts. The framework nanoshell in this work may stimulate the thinking about how to design advanced shell architecture in the core-shell structured photocatalysts to achieve coordinated charge and molecule transport.

## 1. Introduction

Solar-driven photocatalysis is considered to be a promising way to resolve the energy shortage and environmental problems globally by utilizing a sustainable carbon-free energy source instead of conventional fossil energies [1]. It is highly desired for the photocatalysis technology to exploit the visible-light-driven photocatalyst with outstanding performance, which requires the rational structure design based on a specific structure-function relationship. Generally, the photocatalytic process involves the light excitation, separation, transfer, and reaction of photogenerated carriers, together with the transport of reactants and products [2, 3]. Therefore, it becomes one of the most important issues to well coordinate the transport of charge and reactants/products for boosting the photocatalytic activity.

The core-shell structured photocatalyst, which consists of an inner core surrounded by an outer shell, can augment the photocatalytic activity by constructing the versatile heterojunction. Besides that, the integration of core and shell materials can confer tailorable chemical functionalities and physical structural features, thus providing the possibility to regulate the transport process of charge and reactants/products [4, 5]. The accessible active sites at the interface are key to the photocatalytic activity and application, which largely depends on the structure of shell materials. Generally, the porous shell structure can enhance the light harvesting by facilitating the light scattering, shorten the charge transfer distance from inside to outside, and afford the low-resistance diffusion channel for the molecule arriving at the interface. In recent years, the design for the shell materials has attracted increasing attention and various semiconductors have been employed to construct the shell structure so far, such as TiO_2_ [6], SrTiO_3_ [7], g-C_3_N_4_ [8], and MOFs [9], manifesting a tendency from dense to porous materials. Compared with the dense shell, the porous shell holds greater promise owing to the superiority in enabling the unimpeded light capture as well as the free diffusion of reactants/products.

Covalent organic frameworks (COFs), a kind of porous crystalline materials, are precisely integrated through the topology and building block predesign. Featuring structural periodicity, high porosity, and tunable chemistry, COFs have gained intensive research interests in diverse fields including gas storage and separation, adsorption and encapsulation of chemicals, and heterogeneous catalysis [10–12]. Specifically, their extended conjugated network endows them with a new class of photoactive materials, which possess distinct advantages compared with their inorganic and polymer counterparts [13]. Especially, the well-defined modular building blocks along with the rigid framework affords a preorganized transport pathway for photogenerated electrons and holes to drive the reduction or oxidation reaction in the specific structural units separately, decreasing the disordered charge transport. Besides the in-plane mass transport of COFs, their densely aligned *π* columns and arrays over the skeleton can be acted as another prearranged pathway to facilitate the charge transport [14, 15]. Moreover, the ordered pore supplies a transfer channel for reactants and products, which inhibits the reverse reaction caused by the sluggish product migration [16, 17]. Accordingly, the rise of COF materials offers a new opportunity for the rational design and delicate regulation of the shell structure in the core-shell photocatalyst.

Herein, aiming at coordinating the charge transfer and molecule diffusion, the COF nanoshell was first bottom-up synthesized on the surface of cadmium sulfide (CdS) nanospheres to construct independent transfer pathways through the core-shell CdS@COF nanospheres. A typical COF, TPPA, featuring high stability in water, acid, and base arisen from its enol–keto tautomerization structure [18], was *in situ* synthesized through interfacial polymerization using 1, 3, 5-triformylphloroglucinol (TP) and p-phenylenediamine (PA) as monomers, which is different from the postsynthetic construction for COF-based hybrid materials [19]. CdS, one of the most viable candidates for practical application, was exploited as the core due to its appropriate redox potential and narrow bandgap (about 2.4 eV) [20–22]. Well-matched bandgaps between TPPA and CdS, along with the porous TPPA nanoshell structure, are beneficial to the charge transfer and molecule diffusion. The photocatalytic activity of as-prepared core-shell CdS@TPPA nanospheres with thickness-tunable COF nanoshells was evaluated by the hydrogen evolution under visible light irradiation. This study demonstrates the great potential of using the organic framework as a nanoshell for the coordination of charge transfer and molecule diffusion process in photocatalytic applications.

## 2. Results

### 2.1. Preparation and Characterizations of CdS@TPPA Photocatalyst

**Figure 1:**
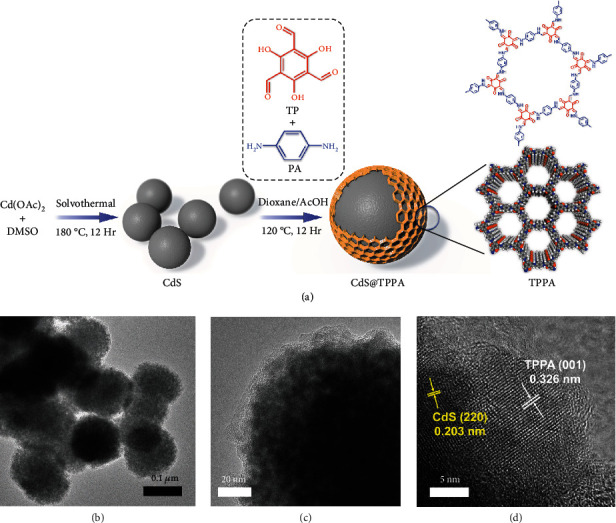
(a) Schematic diagram of the on-surface bottom-up synthesis route for core-shell CdS@TPPA nanospheres. (b) TEM and (c, d) HRTEM images of CdS@TPPA2.

As illustrated in Figure 1(a), the core-shell CdS@TPPA nanospheres were prepared *via* a facile on-surface bottom-up route. In brief, a kind of monomer, PA, is first adsorbed through the electrostatic interaction on the surface of CdS nanospheres to provide the growth sites for TPPA. Then, another monomer, TP, is slowly injected to react with anchored PA molecules *via* the Schiff-base reaction, triggering *in situ* polymerization and growth of amorphous TPPA. The obtained polymers on the surface are subjected to the dynamic covalent chemistry under the solvothermal treatment for a structural transformation from amorphous polymer to crystalline COF [23]. Finally, the core-shell CdS@TPPA nanospheres are acquired, in which the TPPA nanoshell is constructed on the surface of CdS nanospheres. This strategy may afford a generic technology to synthesize the COF-based nanoshell on the surface of inorganic materials for achieving high-efficiency nanostructured photocatalysts.

The morphology and crystalline domains of the synthesized CdS@TPPA samples were identified by transmission electron microscopy (TEM) and high-resolution transmission electron microscopy (HRTEM). Pristine CdS materials exhibit a well-defined spherical structure about 80-120 nm in diameter (supplementary Figure S1). After covering the TPPA shell, the size of core-shell nanospheres changes slightly (Figure 1(b)), indicating the ultrathin shell. A distinct interface in the CdS@TPPA samples with a rough surface can be observed (Figure 1(c)), confirming the formation of the core-shell structure. Moreover, the thickness of TPPA nanoshells can be tuned from 5 ± 1 nm of CdS@TPPA1 to 8 ± 1 nm of CdS@TPPA2 and 11 ± 1 nm of CdS@TPPA3 by varying the amounts of two monomers (supplementary Figure S2). The HRTEM image reveals the fringe spacings of about 0.326 and 0.203 nm, which correspond to the (001) lattice plane of TPPA and the (200) lattice plane of CdS, respectively (Figure 1(d) and supplementary Figure S3). Besides, two lattice planes overlap each other, indicating the formation of the CdS-TPPA heterojunction. The energy dispersive X-ray- (EDX-) mapping illustrates that the Cd and S elements are primarily located at the sample center, while the C, N, and O elements cover a larger region, further demonstrating successful construction of the core-shell CdS@TPPA nanospheres (supplementary Figure S4).

**Figure 2:**
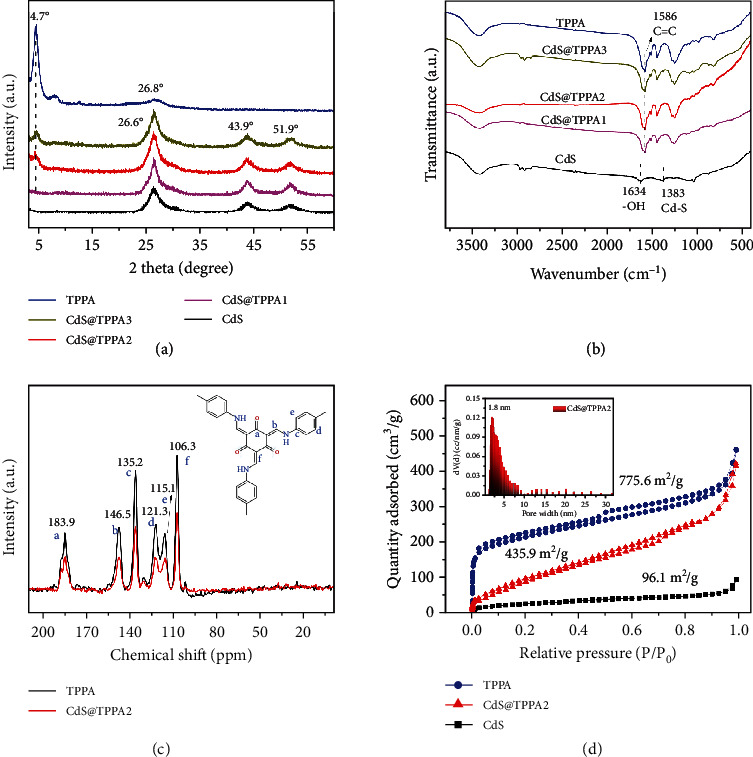
(a) XRD patterns and (b) FTIR spectra of TPPA, CdS@TPPA, and CdS samples. (c) Solid-state ^13^C NMR spectra of TPPA and CdS@TPPA2. (d) N_2_ adsorption-desorption isotherms of TPPA, CdS@TPPA2, and CdS.

The crystalline status and bond formation of CdS@TPPA samples were probed by X-ray diffraction (XRD), Fourier transform infrared spectroscopy (FTIR), and solid-state ^13^C nuclear magnetic resonance (NMR). As displayed in Figure 2(a), three peaks appear at 26.6°, 43.9°, and 51.9° in the XRD pattern of pristine CdS nanospheres, corresponding to the diffraction patterns of the (111), (220), and (311) plane in the cubic-phase CdS crystal (JCPDS: 80-0019), severally [19]. The pristine TPPA curve exhibits two characteristic peaks at 4.7° and 26.8°, separately. The former peak located in the low-angle region is assigned to the (100) plane of TPPA, corresponding to the hexagonal pore about 1.8 nm in diameter calculated from the Bragg equation (*d* = *nλ*/2sin*θ*), which demonstrates the well-preserved long-range ordered structure [24]. The latter peak originates from the (001) plane of TPPA, indicating the formation of two-dimensional COF crystalline with the hexagonal topology [18, 25]. The corresponding *π* − *π* stacking distance is calculated to be about 0.33 nm, which is consistent with the lattice fringe observed in the HRTEM image (Figure 1(d)). Besides three characteristic peaks of CdS, the CdS@TPPA samples exhibit a weak peak at 4.7° related to TPPA, which further verifies the formation of the TPPA shell. In Figure 2(b), the FTIR spectrum of pristine CdS nanospheres shows two weak peaks at 1634 and 1383 cm^−1^, which are attributed to the O–H bending vibration of surface-absorbed water molecules and Cd-S bond, severally [26]. In the FTIR spectrum of pure TPPA, the appearance of the C=C stretching peak at 1586 cm^−1^ and the shift of the C=O stretching peak from 1642 cm^−1^ of TP to 1612 cm^−1^ of TPPA (supplementary Figure S5) reveal the formation of the keto-enamine group [18, 23]. All the CdS@TPPA samples demonstrate similar spectra to pure TPPA due to the weak spectrum of CdS, indicating the formation of the TPPA shell. The chemical structure of CdS@TPPA2 and TPPA was further verified by the solid-state ^13^C NMR spectroscopy (Figure 2(c)). Both of them have a clear signal near 183.9 ppm, suggesting the presence of the carbonyl carbon of the keto form in TPPA [18]. All the above results manifest the chemical structure integrity of COF nanoshells.

Thermogravimetric (TG) analysis was conducted to confirm the thermal stability of the as-prepared samples. The TG curve reveals that CdS@TPPA2 possesses high thermal stability up to 400°C, where the weight loss from 400°C originates from the decomposition of the COF framework [27] (supplementary Figure S6). The permanent porosity of CdS, TPPA, and CdS@TPPA2 was explored by the N_2_ adsorption-desorption measurement. As demonstrated in Figure 2(d), both TPPA and CdS@TPPA2 exhibit typical type I characteristics according to the IUPAC classification, which indicates the micropore presence. Pristine CdS shows the type IV isotherms with the Brunauer-Emmett-Teller (BET) specific surface area of 96.1 m^2^ g^−1^, while the BET specific surface areas of TPPA can reach up to 775.6 m^2^ g^−1^. The core-shell CdS@TPPA2 nanosphere achieves a much larger specific surface area of 435.9 m^2^ g^−1^ in contrast to pristine CdS, which can be attributable to the freshly formed TPPA nanoshell. According to the nonlocal density function theory (NLDFT), the pore-size distribution of CdS@TPPA2 is around 0.8-3.0 nm with the peak maxima at 1.8 nm (inset in Figure 2(d)), as same as the theoretical value of TPPA [18]. This pore size of 1.8 nm, in good accordance with the data calculated from the XRD peak at 4.7° of CdS@TPPA2 (Figure 2(a)), is large enough to accommodate the transfer and diffusion of water, proton, and hydrogen [28–31]. In brief, the core-shell CdS@TPPA nanospheres possess a large specific surface area and well-defined micropore structure, thereby conferring rich active sites for the reaction process, along with abundant channels for the molecule diffusion process [32, 33].

### 2.2. Optical Properties

**Figure 3:**
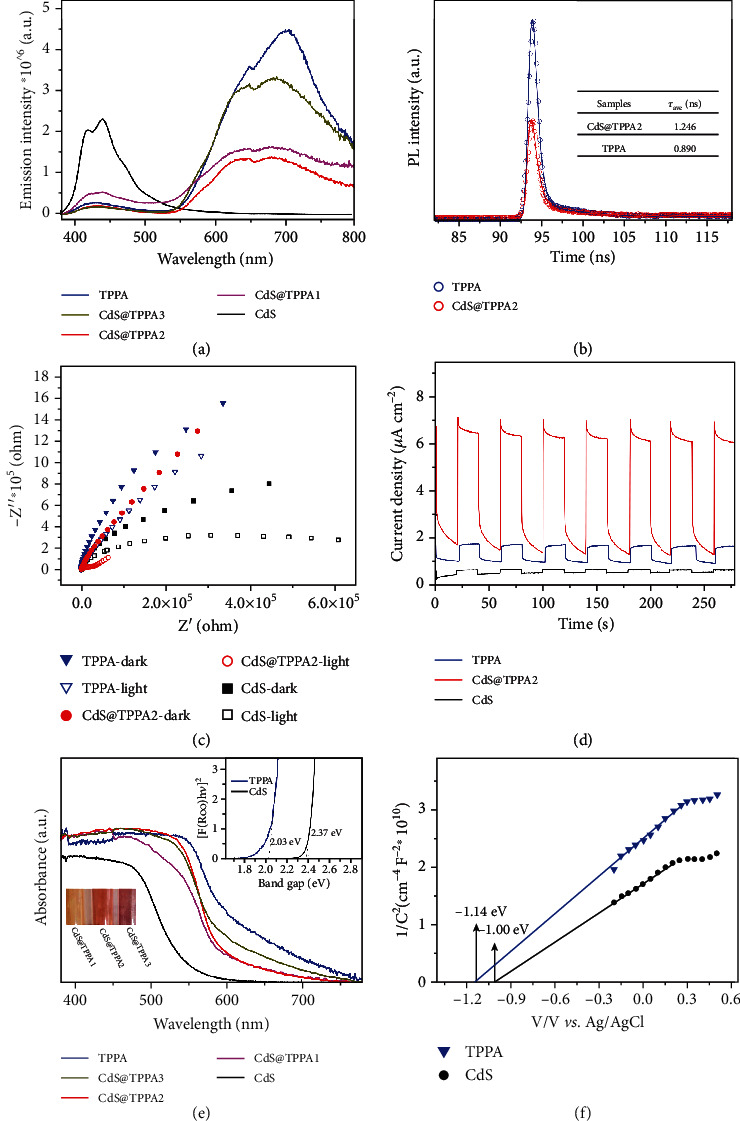
(a) Steady-state PL spectra of TPPA, CdS@TPPA, and CdS with the excitation wavelength of 350 nm. (b) Transient fluorescence lifetime decay profiles of TPPA and CdS@TPPA2. (c) EIS Nyquist plots and (d) photocurrent density curves of TPPA, CdS@TPPA2, and CdS in 0.1 mol L^−1^ Na_2_SO_4_ solution under visible light irradiation. (e) UV-vis DRS of TPPA, CdS@TPPA, and CdS, and the inset is the [*F*(*R*∞)*hv*]^2^*vs.* photon energy plots of CdS and TPPA. (f) Mott-Schottky plots of TPPA and CdS.

The steady-state photoluminescence (PL) spectra and transient fluorescence lifetimes were examined to characterize the charge separation efficiency. As shown in Figure 3(a), the emission peaks of CdS are approximately around 440 nm, while TPPA and the CdS@TPPA samples exhibit dual emission peaks, which is commonly existed in COF due to the excited-state intramolecular proton transfer effect [34, 35]. The emission peaks at 650-680 nm belonging to the keto form of TPPA show lower intensity in the CdS@TPPA samples compared with that of TPPA, suggesting their decreased recombination of photogenerated charge carriers. Moreover, CdS@TPPA2 displays the weakest intensity among them, revealing the crucial role of the TPPA nanoshell thickness in boosting charge separation. It is inferred that the core-shell structure favors facilitating the transfer of photogenerated charges by allowing them to flow along the radial direction, thereby inhibiting the undesirable recombination. However, a migration threshold that is subject to the limited charge mobility [5] gives rise to a higher recombination rate as the thickness exceeds a certain value, as illustrated by the stronger PL intensity of CdS@TPPA3. The fluorescence lifetime decay profile (Figure 3(b)), fitted by the three-exponential fitting method (supplementary Table S1), reveals that the fluorescence lifetimes of TPPA and CdS@TPPA2 are 0.890 and 1.246 ns, respectively. The longer fluorescence lifetime of CdS@TPPA2 compared with TPPA can be attributed to the formed heterojunction structure, manifesting more opportunity for photogenerated electrons transferring to the active sites [36]. Both the decreased PL intensity and prolonged fluorescence lifetime reveal that the charge separation efficiency is greatly improved through constructing the core-shell CdS@TPPA nanospheres. Electrochemical impedance spectroscopy (EIS) was detected to explore the internal resistance in the process of charge transfer. In comparison with those of TPPA and CdS, the Nyquist curve of CdS@TPPA2 presents a smaller semicircular diameter (Figure 3(c)), indicative of the lower internal charge-transfer resistance. Moreover, the semicircle under light is much smaller than that in dark, demonstrating that more electrons produce and transfer to the conduction band (CB) under light illumination [37]. Similarly, CdS@TPPA2 displays a much larger transient photocurrent density (Figure 3(d)), further confirming its lower internal charge-transfer resistance. The UV-vis diffusion reflectance spectroscopy (UV-vis DRS) was conducted to analyze the light capture capacity of TPPA, CdS@TPPA samples, and CdS. As illustrated in Figure 3(e), the adsorption edge of three CdS@TPPA samples is located between those of pristine CdS and TPPA and displays a redshift with the increase of the TPPA nanoshell thickness. According to the Figure 3(e) inset, the corresponding bandgaps of TPPA and CdS are identified as 2.03 and 2.37 eV, respectively. Mott-Schottky plots disclose that the flat band positions of TPPA and CdS are determined to be -1.14 and -1.00 eV *vs.* Ag/AgCl (-0.94 and -0.80 eV *vs.* NHE, pH = 7), separately (Figure 3(f)), and the positive slope reveals that both TPPA and CdS are *n*-type semiconductors. Hence, the CB potential of TPPA and CdS is estimated to be -1.04 and -0.90 eV *vs.* NHE (pH = 7), respectively, which is more negative than the potential for proton reduction (E(H^+^/H_2_) = −0.41 eV*vs.* NHE, pH = 7). Combined with the bandgaps obtained from UV-vis DRS analysis, the valence band (VB) potential of TPPA and CdS is calculated to be 0.99 and 1.47 eV, severally. Such staggered band structure and intimate contact on the interface can result in the formation of the type II heterojunction [38]. The formed heterojunction of the core-shell CdS@TPPA nanospheres drives the charge transfer from one semiconductor to another, which facilitates the spatial isolation of photogenerated electron-hole pairs.

### 2.3. Photocatalytic Performance and Mechanism

**Figure 4:**
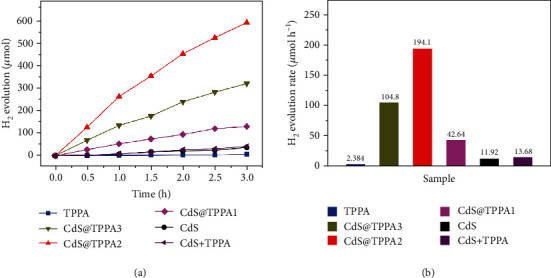
(a) Visible-light-driven H_2_ evolution curves of TPPA, CdS@TPPA samples, CdS, and physical mixture of CdS and TPPA (1 : 1). (b) Corresponding H_2_ evolution rates under visible light.

The visible-light-driven photocatalytic H_2_ evolution as a model reaction was evaluated over TPPA, three CdS@TPPA samples, CdS, physical mixture of CdS and TPPA (1 : 1) with sodium ascorbate (SA) as the hole sacrificial agent and Pt as the cocatalyst. As depicted in Figure 4, the CdS nanosphere displays poor H_2_ evolution rates of 11.92 *μ*mol h^−1^ due to its serious recombination and intrinsic photocorrosion. When coated with the COF nanoshell of 5 ± 1 nm, a much higher photocatalytic activity up to 42.64 *μ*mol h^−1^ was obtained by the sample CdS@TPPA1. Among the prepared samples, CdS@TPPA2 with 8 ± 1 nm nanoshell renders the highest photocatalytic activity of 194.1 *μ*mol h^−1^ (24.3 mmol g^−1^ h^−1^), achieving 80 and 15-fold elevation compared with those of pristine TPPA and CdS, respectively, which is superior to many reported COF-based photocatalysts towards hydrogen evolution. It is noteworthy that a further increase in the nanoshell thickness of 11 ± 1 nm lowers the hydrogen evolution rate to 104.8 *μ*mol h^−1^ for CdS@TPPA3. This result suggests that although the light absorption is extended for a thicker nanoshell, the recombination is less inhibited due to the further stacking decrease in the charge accessible to active sites, which is evident by the higher PL intensity in contrast with that of CdS@TPPA2 (Figure 3(a)). Moreover, the relatively low H_2_ evolution rate of 2.384 *μ*mol h^−1^ for pristine TPPA can be attributable to the high exciton binding energy of all-organic materials and limited exposed sites of bulk structure available for the photocatalytic reaction [39], further manifesting the significant role of the TPPA nanoshell thickness in the photocatalytic activity enhancement.

Moreover, the photocatalytic activity of the physical mixture of CdS and pristine TPPA (1 : 1) is only 13.68 *μ*mol h^−1^, further identifying the effectiveness of the core-shell structure for the activity enhancement. The apparent quantum efficiency (AQE), a crucial parameter for evaluating the apparent efficiency of energy conversion from solar to hydrogen by photocatalysts [40], was measured under monochromatic light with identical reaction conditions. It is calculated that CdS@TPPA2 possesses an AQE of 4.29% at 420 nm (supplementary Figure S7), which is competitive among the COF-based photocatalysts reported (supplementary Table S1). Moreover, the AQE at 455 nm is calculated to be 3.47%, proving that the hydrogen evolution activity is highly dependent on the light-absorption level. Furthermore, the photocatalytic stability of CdS@TPPA2 was assessed by a long-term experiment. The photocatalytic H_2_ evolution rate of CdS@TPPA2 can be maintained at 164.6 *μ*mol h^−1^ for at least 10 h (supplementary Figure S8a), and its core-shell nanosphere structure remains unchanged (supplementary Figure S8b) after 10 h reaction, indicating its strong stability.

The photocatalytic activity of CdS@TPPA2 with different sacrificial reagents like triethanolamine (TEOA) and ascorbic acid (AA) was also explored. The experiment using SA shows a higher H_2_ evolution rate than that using TEOA or AA, which can be attributed to their difference in the reduction ability, indicating the choice significance for the hole sacrificial agent. Considering that the distribution and content of Pt cocatalysts have a great impact on the photocatalytic hydrogen production, the distribution status was confirmed by the TEM, high-angle annular dark-field scanning transmission electron microscopy (HAADF-STEM), and EDX-mapping tests (supplementary Figure S7-S10). As measured by the inductively coupled plasma optical emission spectrometry (ICP-OES) analysis, the obtained Pt amounts in these samples are about 0.99-1.15 wt%, which is lower than the feeding mass ratio but maintains the same level with a slight error. Furthermore, we directly used the Pt-free samples to conduct the photocatalytic reaction. It is noted that the photocatalytic activity of all CdS@TPPA samples is still superior to pristine CdS and TPPA, and similarly, CdS@TPPA2 exhibits the highest H_2_ evolution rate of 7.956 *μ*mol h^−1^ (994.5 *μ*mol g^−1^ h^−1^), which further excludes the activity differences obtained by the Pt content or distribution status.

**Figure 5:**
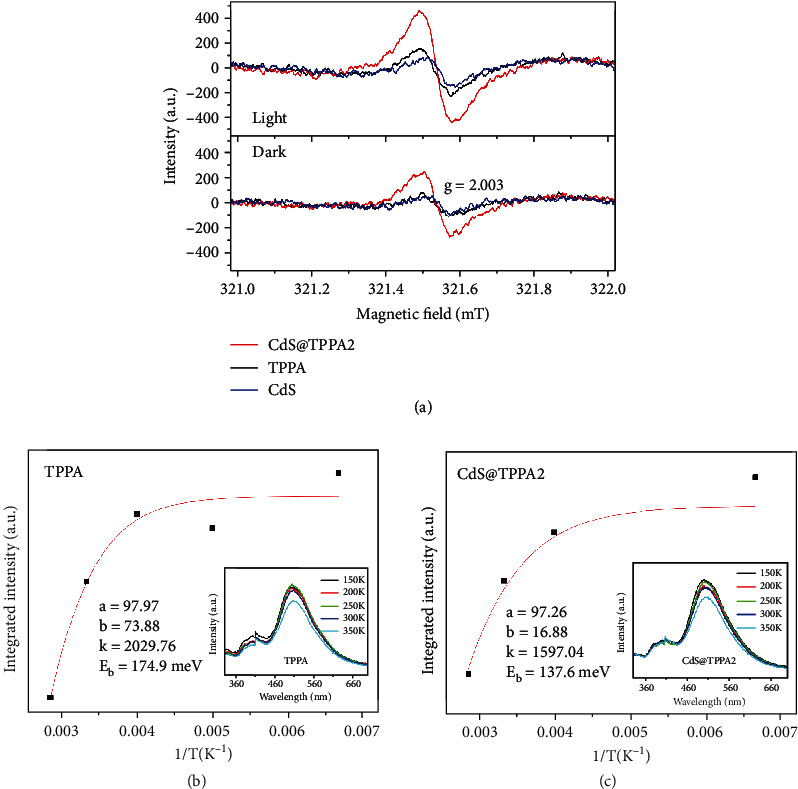
(a) EPR spectra of CdS@TPPA2, TPPA, and CdS under visible light illumination and in dark. Integrated PL emission intensity as a function of temperature (inset: temperature-dependent PL spectra, *λ*_ex_ = 300 nm) of (b) TPPA and (c) CdS@TPPA2.

To further elucidate the charge transfer process, the electron paramagnetic resonance (EPR) signals of CdS@TPPA2, TPPA, and CdS were detected to analyze the unpaired electrons under visible light illumination (*λ* ≥ 420 nm). As depicted in Figure 5(a), all three samples exhibit a single Lorentzian line centered at *g* = 2.003. Compared with those of TPPA and CdS, the EPR signal of CdS@TPPA2 enhances obviously whether under light or dark, originating from a much higher unpaired electron concentration, which is conducive to the proton reduction [41]. Moreover, the EPR signals of all three samples are stronger under visible light irradiation than those in dark, implying an increase in the concentration of unpaired electrons due to the photoinduced charge separation [42]. These results unveil that core-shell CdS@TPPA nanospheres have a more delocalized structure in comparison to pristine TPPA, which can favor the charge migration and then inhibit the charge recombination [43]. The exciton binding energy (*E*_*b*_) of CdS@TPPA2 and TPPA was investigated by the temperature-dependent photoluminescence (Figures 5(b) and 5(c)), because it is crucial for the charge migration and recombination as a key physical parameter of semiconductors. By varying the temperature, the integrated PL peak area can be obtained, and the corresponding *E*_*b*_ can be estimated by fitting these data based on the Arrhenius equation, *I*(*T*) = *I*_0_/1 + *A*exp(−*E*_*b*_/*k*_*B*_*T*). As a result, the *E*_*b*_ of TPPA is derived as 174.9 meV, while that of CdS@TPPA2 decreases to 137.6 meV. Generally, organic semiconductors only have limited photocatalytic activities owing to their undesirably high *E*_*b*_, typically more than 100 meV [39, 44]. Such a distinct reduction of *E*_*b*_ (Δ = 37.3 meV) can lead to a more delocalized charge transfer pathway in the core-shell CdS@TPPA nanosphere, in good accordance with EPR results. Accordingly, we can deduce that the construction of the core-shell CdS@TPPA nanosphere is effective to weaken the *E*_*b*_ of TPPA, thus conferring enhanced charge mobility to the TPPA framework. Hence, the enhanced EPR intensity, as well as the diminished *E*_*b*_, indeed validates the core-shell CdS@TPPA nanosphere towards higher photocatalytic activity than pristine TPPA.

**Scheme 1:**
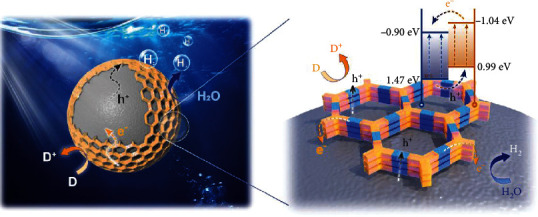
The mechanism of photocatalytic hydrogen evolution over the core-shell CdS@TPPA nanosphere.

Considering all of the above results and discussion, a typical photocatalytic process over the core-shell CdS@TPPA nanosphere can be illustrated in Scheme 1. Under the visible light illumination, the light and reactants (H_2_O/H^+^) can reach the inner core surface through the ordered channels of TPPA nanoshell. Then, both CdS and TPPA are stimulated to generate the electron-hole pairs. Especially for TPPA, its well-defined modular building blocks affords a preorganized transport pathway for electrons and holes to separate spatially, where the electrons flow onto the acceptor units (TP) and the holes left onto the donor units (PA) [17]. Moreover, the type II heterojunction formed between CdS and TPPA can further facilitate the effective charge separation on the interface. Induced by the type II band alignment, the electrons can transfer from the TPPA CB to the CdS CB and reduce the reactants (H_2_O/H^+^) subsequently. Meanwhile, the holes generated by CdS are transferred to the shell along with the PA donors between the stacked layers and finally consumed by the sacrificial agent (D). Finally, the generated products (H_2_) can release outside through the regular pores provided by the COF nanoshell.

Consequently, three independent transport pathways for electron, hole, and reactants/products are established by the porous COF nanoshells during the photocatalytic process, ultimately promote the hydrogen evolution rate efficiently. First, the electron is excited from the PA unit to the TP unit of TPPA, transfers along the TP units of TPPA to reach the CdS CB, and reduces the proton to H_2_. Secondly, the hole shifts from the CdS VB to the TPPA VB, moves along the PA units, and is quenched by the sacrificial agent. Thirdly, the reactants can diffuse easily through the regular pore channel about 1.8 nm in size in the COF structure to the reaction sites on the surface of CdS, and then, the product can also diffuse to the solution effectively *via* the same channel. This kind of core-shell structure consisting of the inorganic nanosphere and COF nanoshell may offer an inspiring idea for the rational design of high-efficiency photocatalysts.

## 3. Conclusion

In summary, the COF nanoshell has been designed and deposited on the surface of CdS nanospheres *via* a bottom-up synthesis method. By modulating the thickness of the TPPA nanoshell, the charge transfer and molecule diffusion process are simultaneously enhanced. With the optimal nanoshell thickness of 8 ± 1 nm, the CdS@TPPA nanosphere acquires an excellent photocatalytic hydrogen evolution rate of 194.1 *μ*mol h^−1^ (24.3 mmol g^−1^ h^−1^, 8 mg) under visible light illumination, approximately 80 and 15-fold elevation compared with those of pristine TPPA and CdS, respectively. The results manifest that the introduction of ordered porous COF structure of TPPA nanoshells creates independent transport pathways for electron, hole, and reactants/products, ultimately enhancing the photocatalytic activity. Considering the vast number of monomers available for organic framework construction and rapid advances in reticular chemistry, the organic framework nanoshell in this work will evolve a powerful platform design. Besides, this work demonstrates how to acquire the coordinated optimization of charge transfer and molecule diffusion by the rational design of the outer shell in the core-shell photocatalyst.

## 4. Materials and Methods

### 4.1. Materials

All chemicals were analytically pure at least and used directly. Cd(OAc)_2_·2H_2_O was supplied by Sigma-Aldrich (St. Louis, USA). 1, 3, 5-Triformylphloroglucinol (TP) and p-Phenylenediamine (PA) were provided by Changchun Third Party Pharmaceutical Technology Co., Ltd. (Changchun, China). Dimethyl sulfoxide (DMSO), N, N-dimethylformamide (DMF), tetrahydrofuran (THF), 1, 4-dioxane, mesitylene, and acetone were purchased from Aladdin Biochemical Technology Co., Ltd. (Shanghai, China). Chloroplatinic acid hexahydrate (H_2_PtCl_6_·6H_2_O), phosphate-buffered saline (PBS, pH = 7.4), sodium ascorbate (SA), triethanolamine (TEOA), and ascorbic acid (AA) were provided by Chemart Chemistry Co. Ltd. (Tianjin, China).

### 4.2. Synthesis of Core-Shell CdS@TPPA Nanospheres

At first, CdS nanospheres were synthesized following the reported method with a minor modification [19]. Specifically, 426.4 mg Cd (OAc)_2_·2H_2_O were dissolved in 160 mL DMSO. After stirring at 800 r min^−1^ for 15 min, the solution was transferred to a Teflon-lined autoclave and heated at 180°C for 12 h. The obtained precipitates were collected by centrifugation, washed three times with acetone and ethanol, and dried at 60°C.

In a typical preparation process of core-shell CdS@TPPA nanospheres, 20 mg synthesized CdS nanospheres were first dispersed into 5 mL 1, 4-dioxane and treated ultrasonically for 20 min. Afterward, 6 mg PA was added, and the resulting dispersion was kept stirring for 1 h at 500 r min^−1^. Meanwhile, 6 mg TP was dispersed into 1 mL 1, 4-dioxane accompanied by ultrasonic treatment for 15 min to get a homogenous solution. The TP solution was injected into the suspension including CdS and PA with a feeding rate of 35 *μ*L min^−1^, followed by adding 1 mL of 3 mol L^−1^ acetic acid. After being stirred for another 30 min, the resulted suspension was transferred to a Schlenk tube immediately and sealed by the typical freeze–vacuum–thaw cycles. Subsequently, the suspension was heated at 120°C for 72 h. The resultant precipitates were collected thereafter, washed with acetone, and dried at 60°C. By varying the amount of TP (3, 6, and 9 mg) and PA (3, 6, and 9 mg), a series of CdS@TPPA samples were obtained, which are marked as CdS@TPPAx (*x* = 1, 2, and 3). For comparison, pristine TPPA was synthesized under the same conditions without adding CdS nanospheres.

### 4.3. Characterizations

TEM images, HAADF-STEM, and EDX-mapping spectra were collected by a JEOL-F200 microscope. XRD patterns were obtained by using the Smartlab instrument (Cu *Kα*, *λ* = 1.5406 Å, Rigaku) over a diffraction angle (2*θ*) range of 3-60° with a step increment of 2*θ* = 0.02° at a rate of 2° min^−1^. FTIR spectra were recorded by a Nicolet 560 spectrometer. Solid-state ^13^C NMR spectra were conducted on a JEOL JNM-ECZ-600R/M1 600 MHz spectrometer. The N_2_ adsorption-desorption isotherms were determined by a Quantachrome surface area analyzer, and the sample powders were first degassed at 150°C for 24 h. The UV-vis DRS was detected by a Hitachi U-3010 spectrometer. Steady-state PL emission spectra and transient fluorescence lifetimes were obtained by a Jobin Yvon Horiba Fluorolog-3 spectrofluorometer. Temperature-dependent PL spectra were processed on an Edinburgh FLS 1000 spectrofluorometer equipped with Oxford Instruments nitrogen cryostat (Optistat DN) for temperature control. EPR spectra measurements were executed on a JES-FA200 X-band instrument. TG analysis was performed on a NETZSCH instrument in the N_2_ atmosphere with a heating rate of 10°C min^−1^. ICP-OES (iCAP 7000 Series, Thermo Fisher Scientific) was conducted to determine the Pt contents in all the samples.

### 4.4. Photocatalytic Activity Measurement

Photocatalytic H_2_ evolution experiment was conducted in an on-line trace gas analysis system (Labsolar 6A, Beijing Perfectlight Technology Co. Ltd.), where the system temperature is maintained by the recirculating cooling water. For each experiment, 8 mg photocatalyst was suspended in a PBS solution containing SA (0.1 g, 50 mL). Using H_2_PtCl_6_ as the precursor, a feeding mass amount of 3 wt% Pt was added to photodeposited as the cocatalyst. This reactor was connected to the vacuum system, and the generated H_2_ was analyzed through a thermal conductivity detector (TCD) on-line gas chromatograph (GC7900, Shanghai Techcomp Co. Ltd.). A 300 W Xe lamp was utilized as the light source and a 420 nm cutoff filter was applied to remove the UV region of the light.

AQE for the photocatalytic H_2_ evolution was assessed by using two LED lamps with *λ* = 420 nm (*E*_0_ = 41.00 mW cm^−2^, *E*_1_ = 30.50 mW cm^−2^, *E*_2_ = 31.30 mW cm^−2^, *E*_3_ = 30.80 mW cm^−2^, *E*_4_ = 28.20 mW cm^−2^) and *λ* = 455 nm LED (*E*_0_ = 31.20 mW cm^−2^, *E*_1_ = 21.20 mW cm^−2^, *E*_2_ = 22.10 mW cm^−2^, *E*_3_ = 17.12 mW cm^−2^, *E*_4_ = 18.25 mW cm^−2^) as the radiation source, respectively. An area of 26 cm^2^ was illuminated, and the light intensity was monitored by a power sensor (CEL-NP2000) at a certain height. AQE was estimated following the method reported in literature [40].

### 4.5. Electrochemical Measurement

The electrochemical measurements were detected by a standard three-electrode cell on a CHI660E electrochemical workstation (Chenhua Instrument, Inc.) in 0.1 mol L^−1^ of aqueous Na_2_SO_4_ solution. The Ag/AgCl, Pt filament, and as-prepared sample were employed as the reference electrode, counter electrode, and working electrode, severally. The working electrode was prepared as follows: first, 2 mg sample was dispersed into 0.5 mL as-prepared Nafion solution to make a suspension; then, the obtained suspension was coated drop by drop onto the fluorine-tin oxide (FTO) glass (2 × 1 cm^2^) and dried at 40°C overnight. The EIS plots were measured at -0.1 V *vs.* Ag/AgCl, while the transient photocurrent was tested under visible light irradiation.

## Data Availability

The data used to support the findings of this study are available from the corresponding author upon request.

## Supplementary Materials

Supplementary Materials Figure S1: TEM image of CdS nanospheres. Figure S2: TEM images and the corresponding thickness profile of (a–c) CdS@TPPA1, (d–f) CdS@TPPA2, and (g–i) CdS@TPPA3 obtained by Digital Micrograph software. Figure S3: the thickness profile of crystal lattice spacing for HRTEM image of (a) CdS and (b) CdS@TPPA2 obtained by Digital Micrograph software. Figure S4: EDX-mapping of CdS@TPPA2. Figure S5: FTIR spectra of TP, PA and TPPA. Figure S6: TG curves of CdS, TPPA and CdS@TPPA2. Figure S7: TEM images of Pt-deposited (a) TPPA, (b) CdS, (c) TPPA1, (d) TPPA2, and (e) TPPA3. Figure S8: (a) HAADF-STEM image and (b–d) EDX-mapping of Pt-deposited TPPA. Figure S9: (a) HAADF-STEM image and (b–d) EDX-mapping of Pt-deposited CdS. Figure S10: (a) High-angle annular dark-field (HAADF) STEM image and (b–f) EDX-mapping of Pt-deposited CdS@TPPA2. Figure S11: (a) visible-light-driven H2 evolution curves of CdS, TPPA, and CdS@TPPA samples without Pt co-catalysts; (b) corresponding H2 evolution rates under visible light. Figure S12: (a) visible-light-driven H2 evolution curves of CdS@TPPA2 with different sacrificial reagents; (b) corresponding H2 evolution rates under visible light. Figure S13: apparent quantum efficiency of CdS@TPPA2 under LED irradiation. Figure S14: (a) long-term H2 evolution curve over CdS@TPPA2 under visible light (≥420 nm); (b) TEM image of CdS@TPPA2 after long-term hydrogen evolution. Table S1: the three-exponential fitting results of fluorescence decay for TPPA and CdS@TPPA2. Table S2: the mass ratio of Pt deposited in different samples calculated from the data of ICP-OES. Table S3: photocatalytic hydrogen evolution activity of the CdS@TPPA nanospheres compared with those of COF-based photocatalysts.
