# Supplementary material for: On-Surface Bottom-Up Construction of COF Nanoshells towards Photocatalytic H2 Production
Source: Research (Wash D C). 2021 Aug 2;2021:9798564. doi: 10.34133/2021/9798564 (PMC8356126; doi:10.34133/2021/9798564)
Supplement: Supplementary Materials — Figure S1: TEM image of CdS nanospheres. Figure S2: TEM images and the corresponding thickness profile of (a–c) CdS@TPPA1, (d–f) CdS@TPPA2, and (g–i) CdS@TPPA3 obtained by Digital Micrograph software. Figure S3: the thickness profile of crystal lattice spacing for HRTEM image of (a) CdS and (b) CdS@TPPA2 obtained by Digital Micrograph software. Figure S4: EDX-mapping of CdS@TPPA2. Figure S5: FTIR spectra of TP, PA and TPPA. Figure S6: TG curves of CdS, TPPA and CdS@TPPA2. Figure S7: TEM images of Pt-deposited (a) TPPA, (b) CdS, (c) TPPA1, (d) TPPA2, and (e) TPPA3. Figure S8: (a) HAADF-STEM image and (b–d) EDX-mapping of Pt-deposited TPPA. Figure S9: (a) HAADF-STEM image and (b–d) EDX-mapping of Pt-deposited CdS. Figure S10: (a) High-angle annular dark-field (HAADF) STEM image and (b–f) EDX-mapping of Pt-deposited CdS@TPPA2. Figure S11: (a) visible-light-driven H2 evolution curves of CdS, TPPA, and CdS@TPPA samples without Pt co-catalysts; (b) corresponding H2 evolution rates under visible light. Figure S12: (a) visible-light-driven H2 evolution curves of CdS@TPPA2 with different sacrificial reagents; (b) corresponding H2 evolution rates under visible light. Figure S13: apparent quantum efficiency of CdS@TPPA2 under LED irradiation. Figure S14: (a) long-term H2 evolution curve over CdS@TPPA2 under visible light (≥420 nm); (b) TEM image of CdS@TPPA2 after long-term hydrogen evolution. Table S1: the three-exponential fitting results of fluorescence decay for TPPA and CdS@TPPA2. Table S2: the mass ratio of Pt deposited in different samples calculated from the data of ICP-OES. Table S3: photocatalytic hydrogen evolution activity of the CdS@TPPA nanospheres compared with those of COF-based photocatalysts. [file 9798564.f1.pdf]

Title

On-surface Bottom-up Construction of COF nanoshells towards Photocatalytic H_2 Production

Authors

Yao Chen[1,4], Dong Yang[2,5], Yuchen Gao[1,4], Runlai Li[3], Ke An[1,4], Wenjing Wang [1,2,5], Zhanfeng Zhao[1,4], Xin Xin[1,2,4], Hanjie Ren[1,4], Zhongyi Jiang*[,1,4,6]

Affiliations

[1] Key Laboratory for Green Chemical Technology, School of Chemical Engineering and Technology, Tianjin University, Tianjin 300072, China

[2] Key Laboratory of Systems Bioengineering of Ministry of Education, School of Chemical Engineering and Technology, Tianjin University, Tianjin 300072, China

[3] College of Polymer Science and Engineering, Sichuan University, Chengdu 610065, China

[4] Collaborative Innovation Center of Chemical Science and Engineering (Tianjin), Tianjin 300072, China

[5] School of Environmental Science and Engineering, Tianjin University, Tianjin 300072, China

[6] Joint School of National University of Singapore and Tianjin University, International Campus of Tianjin University, Binhai New City, Fuzhou 350207, China

Correspondence should be addressed to Zhongyi Jiang; zhyjiang@tju.edu.cn

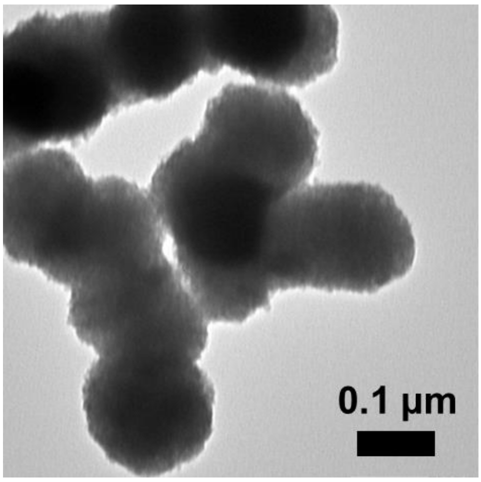

FIGURE S1: TEM image of CdS nanospheres.

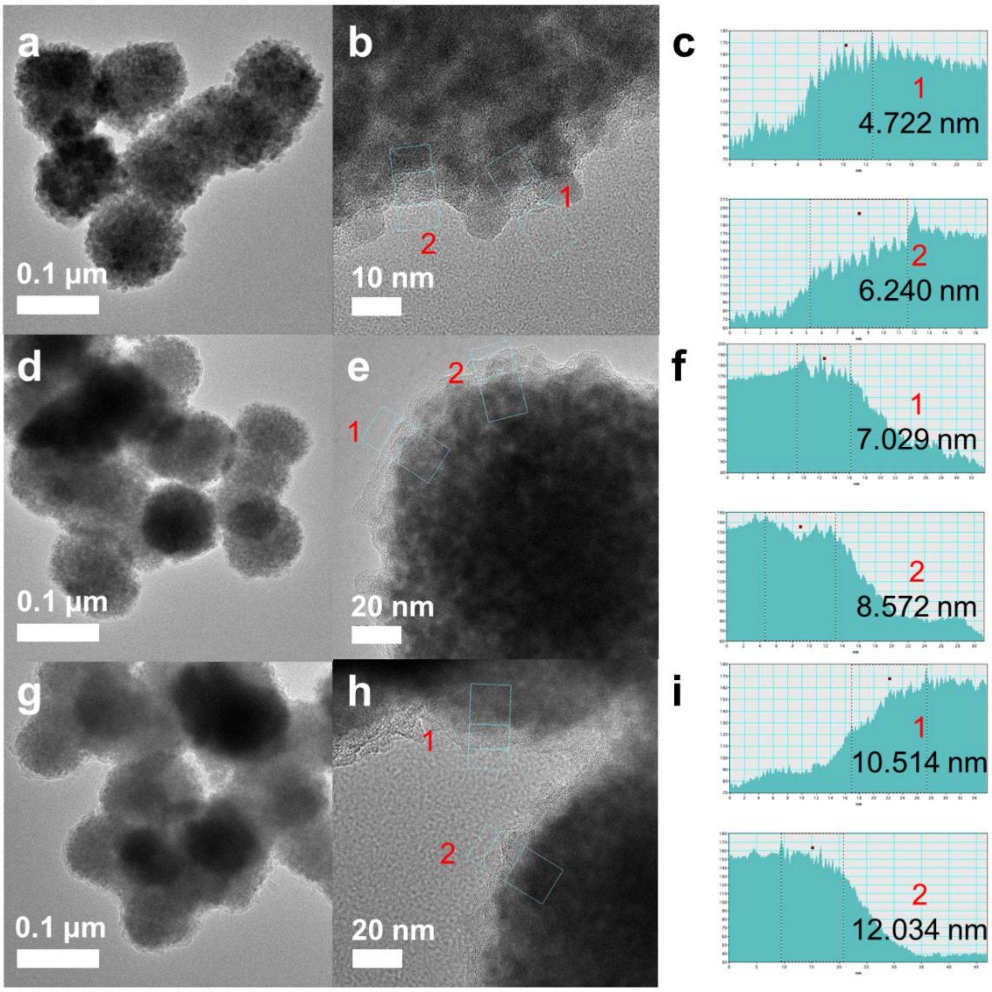

FIGURE S2: TEM images and the corresponding thickness profile of (a-c) CdS@TPPA1, (d-f) CdS@TPPA2 and (g-i) CdS@TPPA3 obtained by Digital Micrograph software.

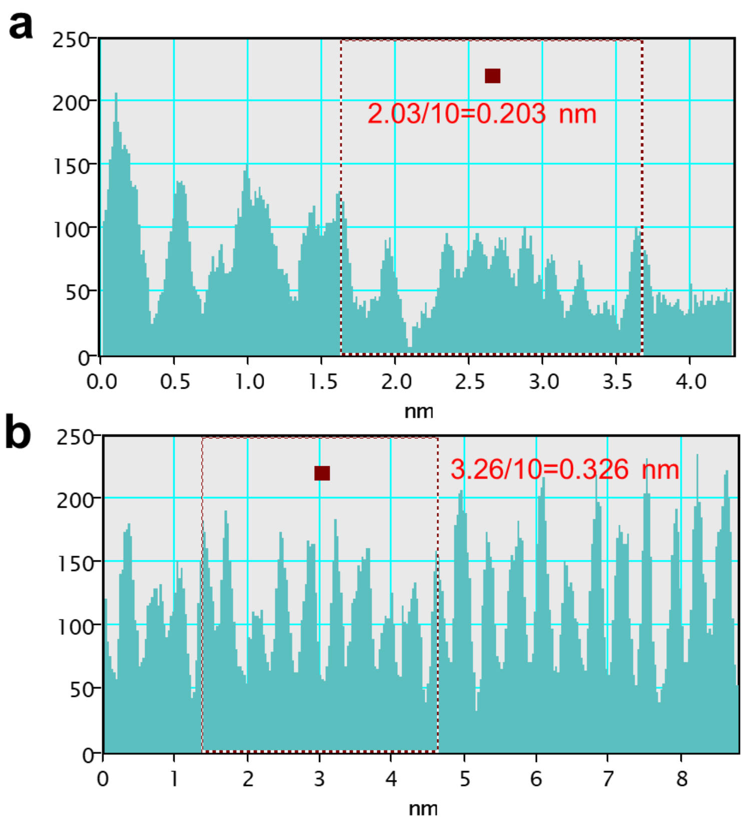

FIGURE S3: The thickness profile of crystal lattice spacing for HRTEM image of (a) CdS and (b) CdS@TPPA2 obtained by Digital Micrograph software.

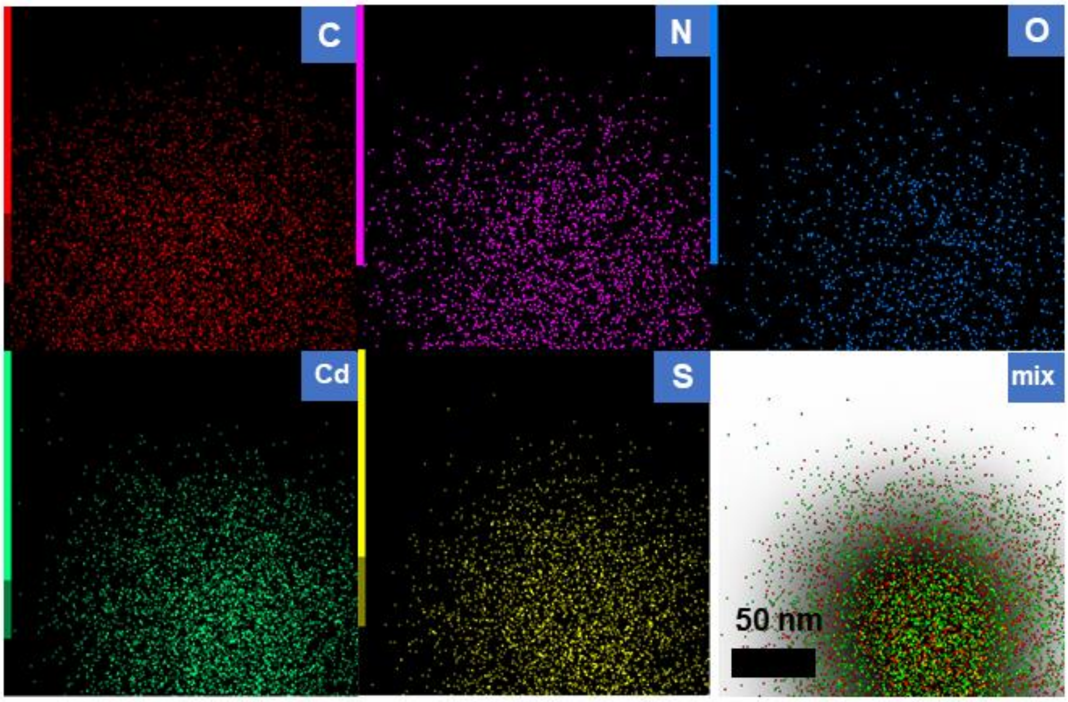

FIGURE S4: EDX-mapping of CdS@TPPA2.

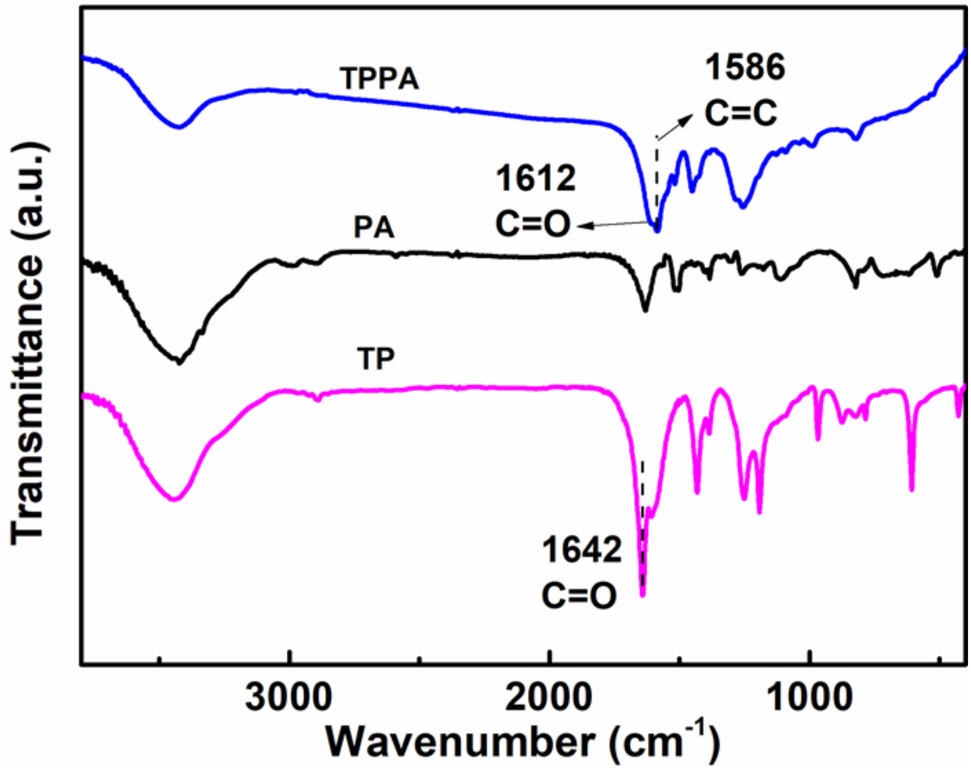

FIGURE S5: FTIR spectra of TP, PA and TPPA.

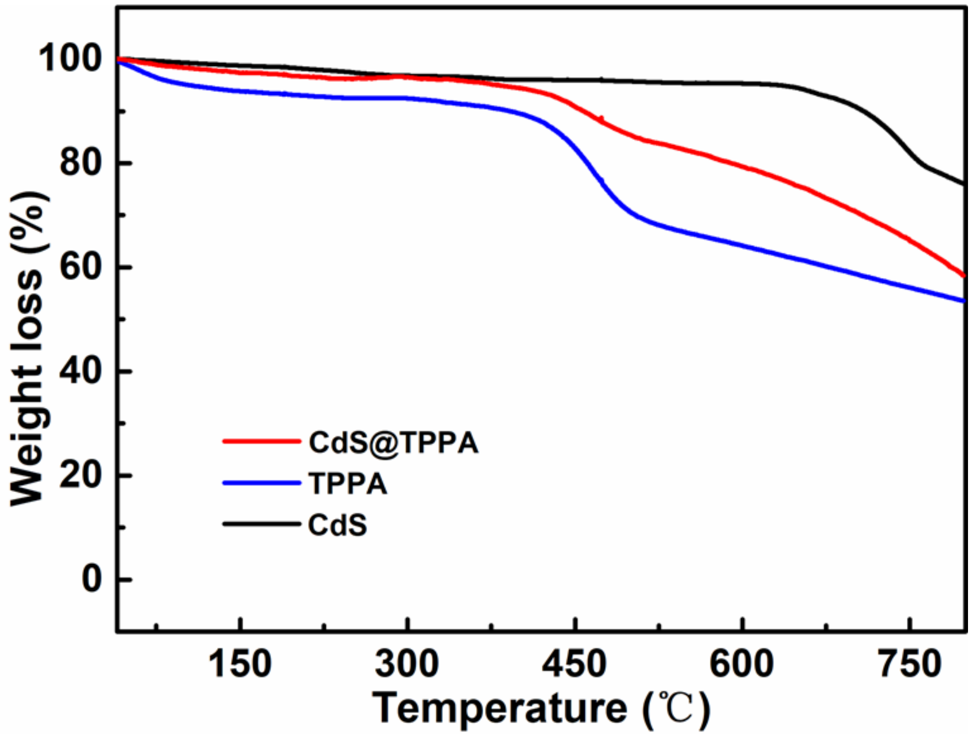

FIGURE S6: TG curves of CdS, TPPA and CdS@TPPA2.

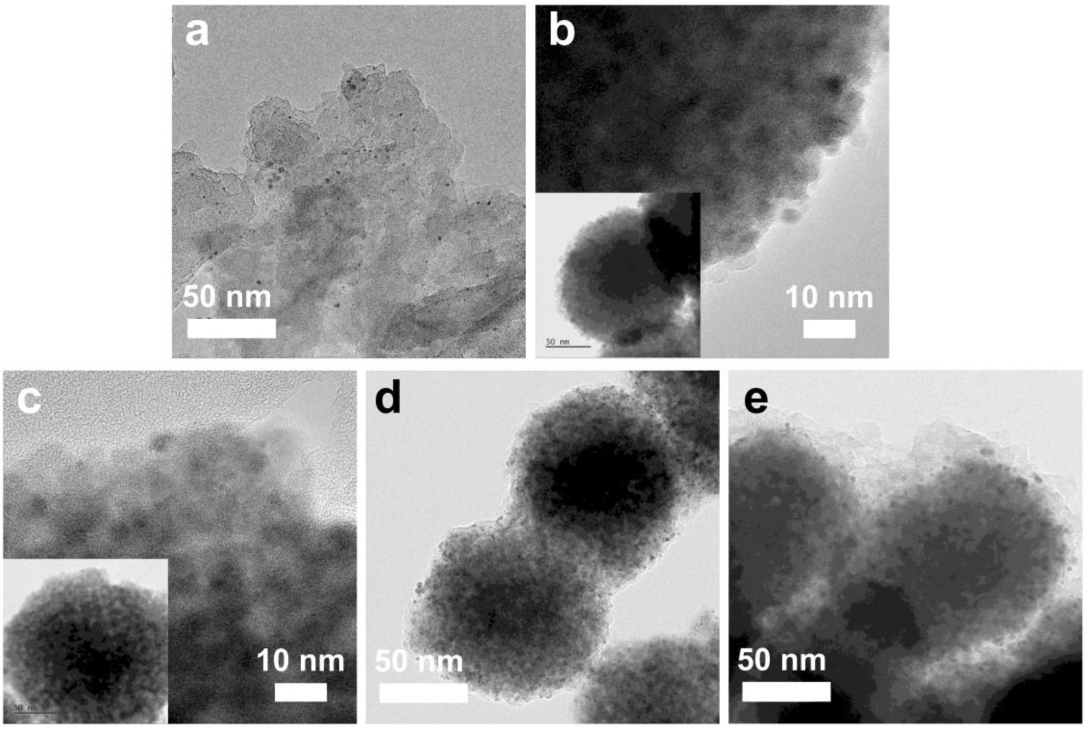

FIGURE S7: TEM images of Pt-deposited (a) TPPA, (b) CdS, (c) TPPA1, (d) TPPA2 and (e) TPPA3.

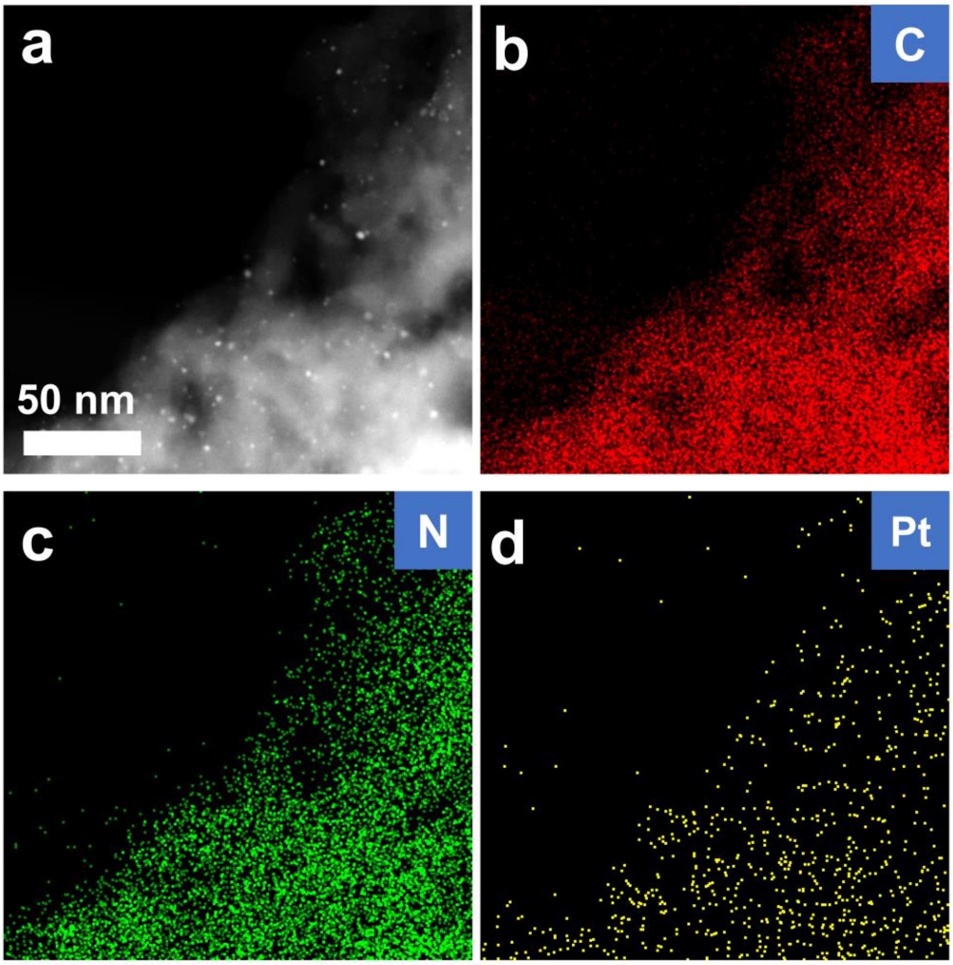

FIGURE S8: (a) HAADF-STEM image and (b-d) EDX-mapping of Pt-deposited TPPA.

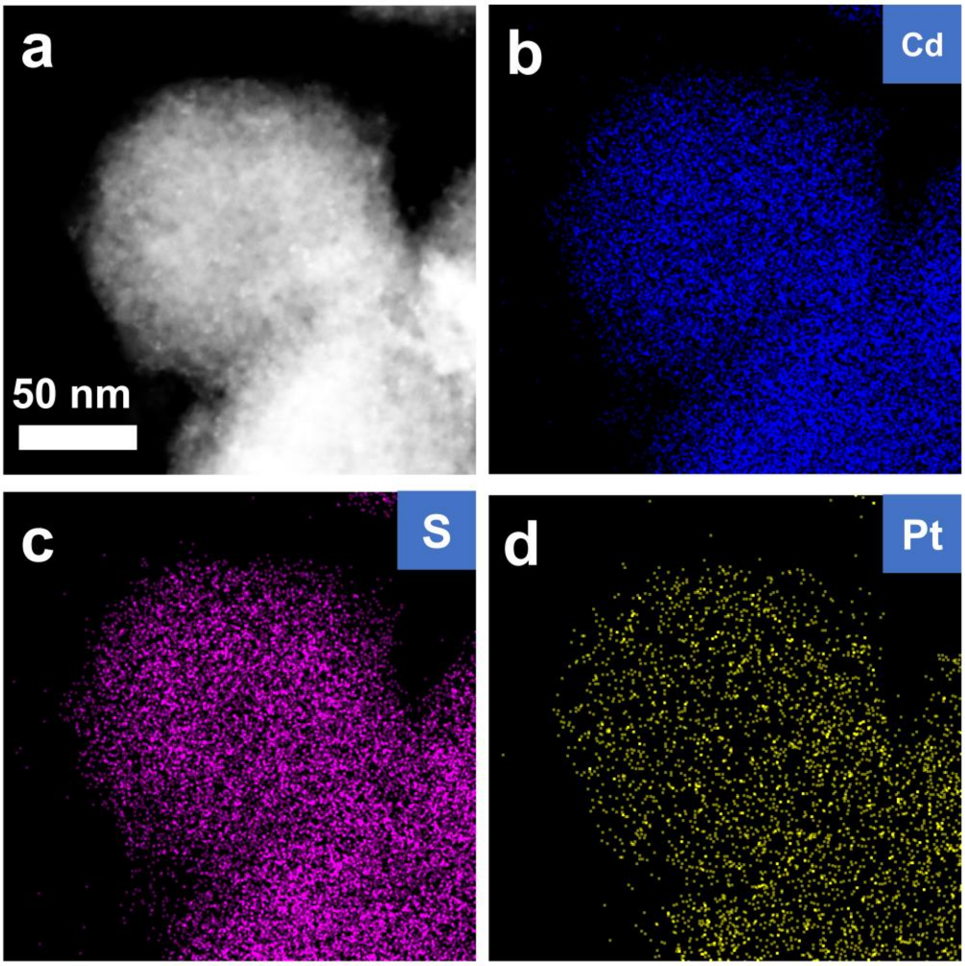

FIGURE S9: (a) HAADF-STEM image and (b-d) EDX-mapping of Pt-deposited CdS.

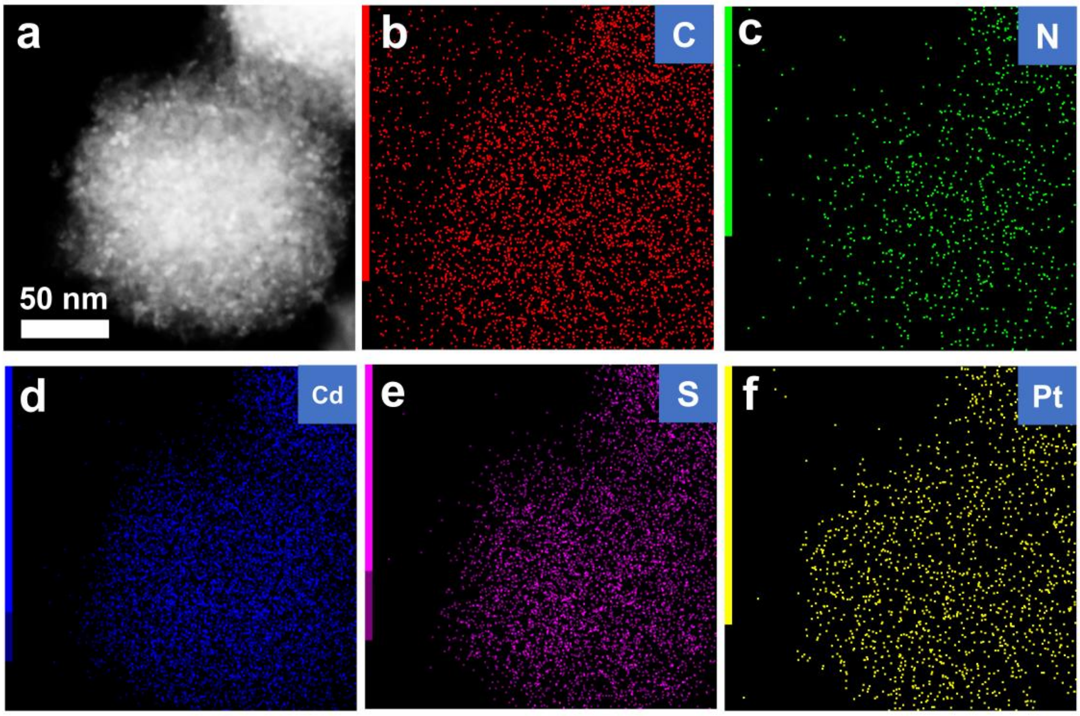

FIGURE S10: (a) High-angle annular dark-field (HAADF) STEM image and (b-f) EDX-mapping of Pt-deposited CdS@TPPA2.

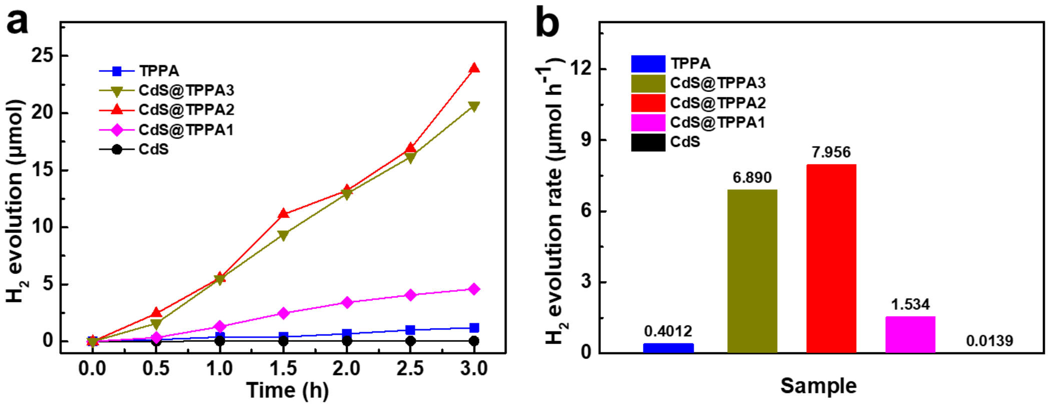

FIGURE S11: (a) Visible-light-driven H_2 evolution curves of CdS, TPPA and CdS@TPPA samples without Pt co-catalysts; (b) Corresponding H_2 evolution rates under visible light.

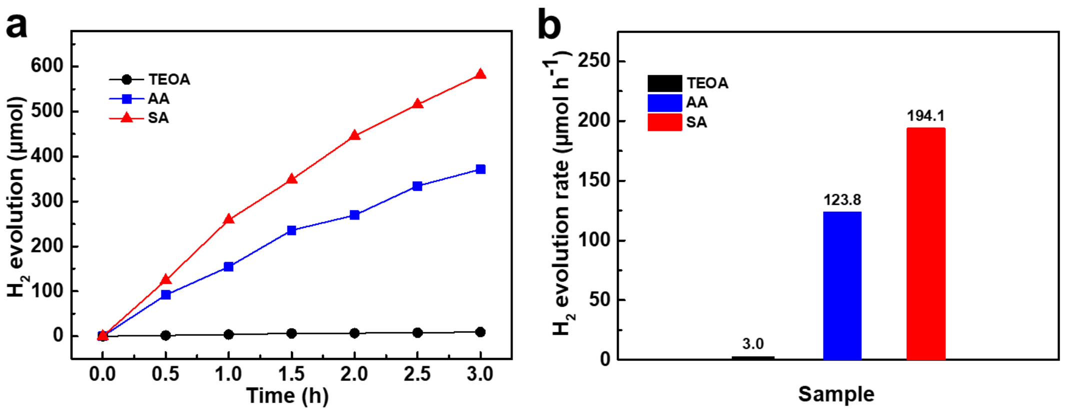

FIGURE S12: (a) Visible-light-driven H2 evolution curves of CdS@TPPA2 with different sacrificial reagents; (b) Corresponding H2 evolution rates under visible light.

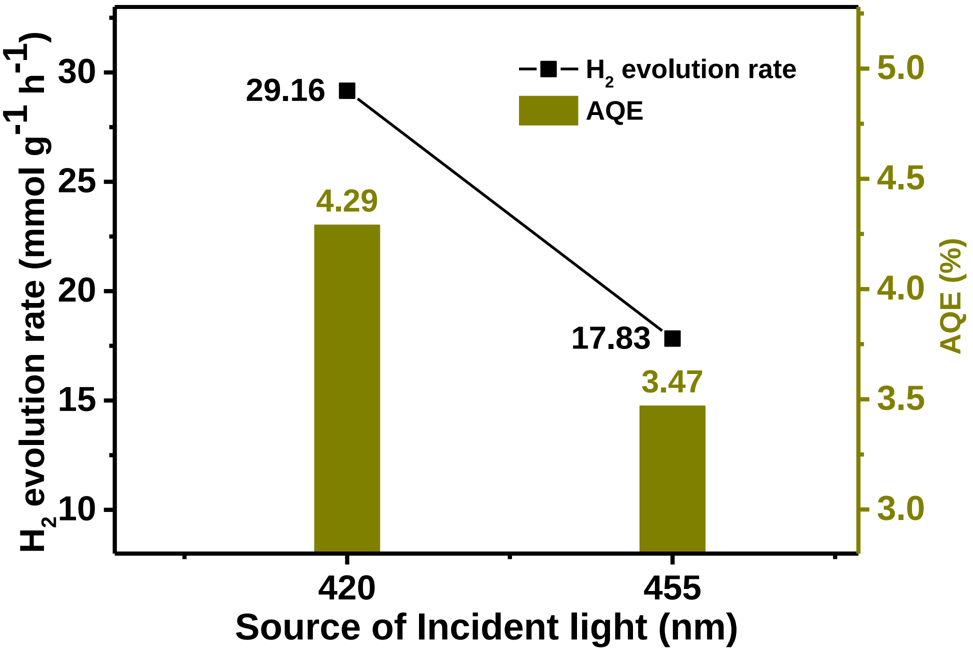

FIGURE S13: Apparent quantum efficiency of CdS@TPPA2 under LED irradiation.

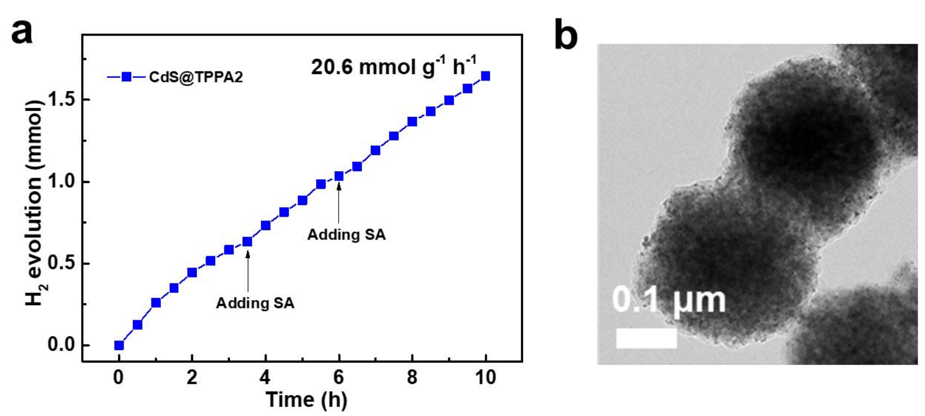

FIGURE S14: (a) Long-term H_2 evolution curve over CdS@TPPA2 under visible light (≥420 nm). (b) TEM image of CdS@TPPA2 after long-term hydrogen evolution

TABLE S1: The three-exponential fitting results of fluorescence decay for TPPA and CdS@TPPA2.

**Samples**	**A1**	**τ1 (ns)**	**A2**	**τ2 (ns)**	**A3**	**τ3 (ns)**	**τ (ns)**
TPPA	0.03	5.478±0.221	0.01	49.242±3.172	0.96	0.505±0.006	0.890
CdS@TPPA2	0.04	5.691±0.386	0.01	37.256±4.478	0.95	0.711±0.009	1.246

TABLE S2: The mass ratio of Pt deposited in different samples calculated from the data of ICP-OES.

**Photocatalyst**	**wt% of Pt**
CdS	1.15
CdS@TPPA1	1.11
CdS@TPPA2	1.08
CdS@TPPA3	1.10
TPPA	0.99

TABLE S3: Photocatalytic hydrogen evolution activity of the CdS@TPPA nanospheres compared with those of COF-based photocatalysts.

Catalyst	Cocatalyst/ photosens -itizer	Sacrificial agent	Solvent	Light source	Activity (mmol g^{-1} h^{-1})	AQE	Ref
TFPT-COF	Pt	TEOA[a]	H_2O	≥420 nm, 300 W	1.97	2.2% (400 nm)	[1]
N_3-COF	Pt	TEOA	PBS	≥420 nm, 300 W	1.70	0.15% (400 nm)	[2]
TP-BDDA-COF	Pt	TEOA	H_2O	≥ 395 nm, 300 W	0.324	1.30% (420 nm)	[3]
A-TEBPY-COF	Pt	TEOA	PBS	AM 1.5	0.0980	-	[4]
PTP-COF	Pt	TEOA	PBS	AM 1.5	0.0838	-	[5]
g-$C_{40}N_3$-COF	Pt	TEOA	H_2O	≥ 420 nm, 300 W	4.12	4.84% (420 nm)	[6]
FS-COF	Pt	SA	H_2O	≥ 420 nm, 300 W	10.1	3.2% (420 nm)	[7]
TpDTz-COF	NiME	TEOA	H_2O	AM 1.5	0.941	0.2% (400 nm)	[8]
TpPa-1-COF	MoS_2	SA	H_2O	≥ 420 nm, 300 W	5.59	0.76% (420 nm)	[9]
NUS55	$[Co(bpy)_3]Cl_2$	TEA[b]	EtOH[c]/ H_2O	≥ 420 nm, 300 W	2.48	1.55% (450 nm)	[10]
TiO_2-TpPa-1-COF	Pt	SA	PBS	≥420 nm, 300 W	11.2	7.6% (420 nm)	[11]
Tp-2C/BPy^{2+}-COF	Pt	SA	H_2O	≥420 nm, 300 W	34.6	6.93% (420 nm)	[12]
NH_2-UiO-66/TpPa-1	Pt	SA	PBS	≥420 nm, 300 W	23.4	-	[13]
α-Fe_2O_3/TpPa-2-COF	-	SA	PBS	≥420 nm, 300 W	3.77	0.137% (450 nm)	[14]
TiO_2@BpZn-COP	Pt	TEOA	H_2O	≥ 420 nm, 300 W	1.33	2.5% (420 nm)	[15]
Mo_3S_{13}@EB-COF	$Ru(bpy)_3$	SA	DMF /H_2O	≥ 420 nm, 300 W	13.2	3.65% (450 nm), 2.57% (435 nm)	[16]
CdS/TPPA-2	Pt	LA[d]	H_2O	≥ 420 nm, 400 W	3.7	4.2% (420 nm)	[17]
NH_2-UiO-66@TFPT-DETH	Pt	SA	PBS	≥420 nm, 300 W	7.18	1.11% (420 nm)	[18]
CdS@TPPA	Pt	SA	PBS	≥420 nm, 300 W	24.3	3.47% (455 nm), 4.29% (420 nm)	This work

[a] TEOA is Triethanolamine.

[b] TEA is triethylamine.

[c] EtOH is ethanol.

[d] LA is lactic acid.

REFERENCES

[1] L. Stegbauer, K. Schwinghammer and B. V. Lotsch, "A Hydrazone-Based Covalent Organic Framework for Photocatalytic Hydrogen Production", Chemical Science, vol. 5, no. 7, pp. 2789-2793, 2014.

[2] V. S. Vyas, F. Haase, L. Stegbauer et al., "A Tunable Azine Covalent Organic Framework Platform for Visible Light-Induced Hydrogen Generation", Nature Communications, vol. 6, pp. 8508-8516, 2015.

[3] P. Pachfule, A. Acharjya, J. Roeser et al., "Diacetylene Functionalized Covalent Organic Framework (COF) for Photocatalytic Hydrogen Generation", Journal of the American Chemical Society, vol. 140, no. 4, pp. 1423-1427, 2018.

[4] L. Stegbauer, S. Zech, G. Savasci et al., "Tailor-Made Photoconductive Pyrene-Based Covalent Organic Frameworks for Visible-Light Driven Hydrogen Generation", Advanced Energy Materials, vol. 8, no. 24, pp. 1703278-1703285, 2018.

[5] F. Haase, T. Banerjee, G. Savasci et al., "Structure-Property-Activity Relationships in a Pyridine Containing Azine-Linked Covalent Organic Framework for Photocatalytic Hydrogen Evolution", Faraday Discussions, vol. 201, pp. 247-264, 2017.

[6] S. Bi, C. Yang, W. Zhang et al., "Two-Dimensional Semiconducting Covalent Organic Frameworks Via Condensation at Arylmethyl Carbon Atoms", Nature Communications, vol. 10, no. 1, pp. 2467-2476, 2019.

[7] X. Wang, L. Chen, S. Y. Chong et al., "Sulfone-Containing Covalent Organic Frameworks for Photocatalytic Hydrogen Evolution from Water", Nature Chemistry, vol. 10, no. 12, pp. 1180-1189, 2018.

[8] B. P. Biswal, H. A. Vignolo-Gonzalez, T. Banerjee et al., "Sustained Solar H2 Evolution from a Thiazolo[5,4-D]Thiazole-Bridged Covalent Organic Framework and Nickel-Thiolate Cluster in Water", Journal of the American Chemical Society, vol. 141, no. 28, pp. 11082-11092, 2019.

[9] M. Y. Gao, C. C. Li, H. L. Tang et al., "Boosting Visible-Light-Driven Hydrogen Evolution of Covalent Organic Frameworks through Compositing with MoS2: A Promising Candidate for Noble-Metal-Free Photocatalysts", Journal of Materials Chemistry A, vol. 7, no. 35, pp. 20193-20200, 2019.

[10] J. Wang, J. Zhang, S. B. Peh et al., "Cobalt-Containing Covalent Organic Frameworks for Visible Light-Driven Hydrogen Evolution", Science China-Chemistry, vol. 63, no. 2, pp. 192-197, 2020.

[11] C. C. Li, M. Y. Gao, X. J. Sun et al., "Rational Combination of Covalent-Organic Framework and Nano TiO2 by Covalent Bonds to Realize Dramatically Enhanced Photocatalytic Activity", Applied Catalysis B: Environmental, vol. 266, pp. 118586, 2020.

[12] Z. Mi, T. Zhou, W. Weng et al., "Covalent Organic Frameworks Enabling Site-Isolation of Viologen-Derived Electron Transfer Mediators for Stable

Photocatalytic Hydrogen Evolution", Angewandte Chemie-International Edition, 2021.

[13] F. M. Zhang, J. L. Sheng, Z. D. Yang et al., "Rational Design of MOF/COF Hybrid Materials for Photocatalytic H2 Evolution in the Presence of Sacrificial Electron Donors", Angewandte Chemie-International Edition, vol. 57, no. 37, pp. 12106-12110, 2018.

[14] Y. P. Zhang, H. L. Tang, H. Dong et al., "Covalent-Organic Framework Based Z-Scheme Heterostructured Noble-Metal-Free Photocatalysts for Visible-Light-Driven Hydrogen Evolution", Journal of Materials Chemistry A, vol. 8, no. 8, pp. 4334-4340, 2020.

[15] Q. Q. Yang, P. Peng and Z. H. Xiang, "Covalent Organic Polymer Modified TiO2 Nanosheets as Highly Efficient Photocatalysts for Hydrogen Generation", Chemical Engineering Science, vol. 162, pp. 33-40, 2017.

[16] Y. J. Cheng, R. Wang, S. Wang et al., "Encapsulating Mo3s13 (2-) Clusters in Cationic Covalent Organic Frameworks: Enhancing Stability and Recyclability by Converting a Homogeneous Photocatalyst to a Heterogeneous Photocatalyst", Chemical Communications, vol. 54, no. 96, pp. 13563-13566, 2018.

[17] J. Thote, H. B. Aiyappa, A. Deshpande et al., "A Covalent Organic Framework-Cadmium Sulfide Hybrid as a Prototype Photocatalyst for

Visible-Light-Driven Hydrogen Production", *Chemistry - A European Journal*, vol. 20, no. 48, pp. 15961-15965, 2014.

[18] Y. Chen, D. Yang, B. Shi et al., "In Situ Construction of Hydrazone-Linked COF-Based Core-Shell Hetero-Frameworks for Enhanced Photocatalytic Hydrogen Evolution", Journal of Materials Chemistry A, vol. 8, no. 16, pp. 7724-7732, 2020.
